# Exploring the Gastrointestinal Microbiome of Eurasian Griffon Vultures (*Gyps fulvus*) Under Rehabilitation in Portugal and Their Potential Role as Reservoirs of Human and Animal Pathogens

**DOI:** 10.3390/vetsci11120622

**Published:** 2024-12-04

**Authors:** Mariana Limede Nascimento, Isa Serrano, Eva Cunha, Filipa Lopes, Pedro Pascoal, Marcelo Pereira, Mónica Nunes, Luís Tavares, Ricardo Dias, Manuela Oliveira

**Affiliations:** 1 CIISA-Centre for Interdisciplinary Research in Animal Health, Faculty of Veterinary Medicine, University of Lisbon, Avenida da Universidade Técnica, 1300-477 Lisboa, Portugal; mariananascimento6@hotmail.com (M.L.N.); evacunha@fmv.ulisboa.pt (E.C.); ltavares@fmv.ulisboa.pt (L.T.); moliveira@fmv.ulisboa.pt (M.O.); 2 Associate Laboratory for Animal and Veterinary Sciences (AL4AnimalS), 1300-477 Lisboa, Portugal; 3 CERAS-Wildlife Study and Rehabilitation Centre, Quercus ANCN, Rua Tenente Valadim 19, 6000-284 Castelo Branco, Portugal; ana.f.lopes@cm-lisboa.pt; 4 cE3c—Centre for Ecology, Evolution and Environmental Changes & CHANGE—Global Change and Sustainability Institute, Faculdade de Ciências, Universidade de Lisboa, Campo Grande, 1749-016 Lisboa, Portugal; pfpascoal@ciencias.ulisboa.pt (P.P.); msnunes@ciencias.ulisboa.pt (M.N.); rpdias@ciencias.ulisboa.pt (R.D.); 5 BioISI-Biosystems and Integrative Sciences Institute, Faculty of Sciences, University of Lisbon, 1749-016 Lisboa, Portugal; mlpereira@fc.ul.pt

**Keywords:** core microbiome, gastrointestinal microbiome, *Gyps fulvus*, human pathogens, animal pathogens, public health, rehabilitation centers, scavengers

## Abstract

The Eurasian griffon vulture (*Gyps fulvus*), a widely distributed scavenger, plays a crucial role in ecosystem health by consuming decomposing carcasses. Scavengers have adapted to avoid disease from the rotting carrion they feed on, probably through a specialized gut microbiome. This study aimed to characterize the gut microbiome of *G. fulvus* (n = 8) present in two rehabilitation centers in mainland Portugal and evaluate their potential as reservoirs of pathogens. Samples were studied through high-throughput 16S rDNA amplicon sequencing of the hypervariable V3-V4 regions and further analyzed using the Qiime2 bioinformatics platform. Our results showed that factors such as sex, location, and time of sampling did not significantly affect the gut microbiome of the griffon vulture. Its composition was highly similar to that of phylogenetically closed animals. However, several potential human and veterinary pathogens were identified. In conclusion, the gut microbiome of *Gyps fulvus* in rehabilitation centers is not significantly altered by stress associated with captivity. Its composition is similar to that of other vultures and scavengers due to their identic diet and needs, suggesting a well-conserved functional gut microbiome, which seems to be influenced by season. The potential risks posed by the identified pathogens to humans and other animals should be further investigated.

## Simple Summary

The Eurasian griffon vulture (*Gyps fulvus*) is a widely distributed scavenger that plays a crucial role in ecosystems by consuming decomposing carcasses and removing hazardous organic matter. This study aimed to analyze the microbial community in the gut of *G. fulvus* residing in wildlife rehabilitation centers in mainland Portugal. The results indicated that this microbial community was not significantly affected by stress associated with captivity, and its composition was similar to that of other vultures and scavengers due to their shared diet and ecological needs. We also recommend further research into the potential risks to humans and other animals posed by several human and veterinary pathogens identified in the gut of *G. fulvus*.

## 1. Introduction

Eurasian griffon vultures (*Gyps fulvus*, Accipitriformes Order, Accipitridae Family) are a group of Old World vultures that forage through large areas of Africa, Asia, and Europe [1]. The global populations of Eurasian griffon vultures have suffered serious and long-term declines throughout decades [1], but nowadays, the population trend is positive [2]. These declines have been associated with several causes. Worldwide, vulture mortality is mainly caused by the consumption of wildlife carcasses that have ingested poisoned baits [3,4,5,6], while in Europe, the prohibition of the maintenance of livestock carcasses in the field after the outbreak of Creutzfeldt-Jakob disease led to a deprivation of carrion availability, which strongly affected scavenger populations [7,8,9]. Although EU rules were improved with EC 322/2003, EC 830/2005, and EC 142/2011 [9], there is still a lack of carrion supply in scavenger feeding stations in Portugal.

*G. fulvus* is widely distributed across the Iberian Peninsula, except for the south of Valencia, Galicia, the east of Andalucia, and the Atlantic corridor in Portugal (Bachmayr et al., 2005), with the Portuguese and Spanish populations being connected [10,11,12]. According to the census performed in 2018, Spain holds 90% of the European population of *G. fulvus*, with a total of approximately 30.946 reproductive pairs [10]. In Portugal, nearly 1000 reproductive pairs of *G. fulvus* are present [2,13] (https://www.listavermelhadasaves.pt/atlas/gyps-fulvus/ accessed on 18 November 2024), mainly distributed over large river basins, especially in the Tejo River, and their affluents. In this Iberian country, *G. fulvus* is classified as being of Least Concern [14] (https://listavermelhadasaves.pt/lista-vermelha/ accessed on 18 November 2024) and is protected according to the EU Birds Directive 2009/147/EC [15].

Vultures are the only vertebrates that are obligate scavengers [16,17,18]. They feed on one- to five-day-old carcasses, mainly from livestock species, such as sheep, goats, cattle, donkeys, and occasionally horses [11], but also from wild species. Vultures contribute to the removal of hazardous decaying bodies [19], having a major role in regulating the spread of pathogenic agents and diseases and contributing to nutrient recycling [20,21]. The defense mechanisms of scavengers against the bacterial microorganisms and toxins present in the rotten carrion they consume include a specialized gastrointestinal tract (GIT) microbiome [22] aided by a gastric and digestive system with low pH and a specific oxygen concentration [23].

As observed in vertebrates, the avian microbiome is highly dynamic, and both extrinsic (e.g., diet, environment) and intrinsic factors (e.g., age, health) influence the composition and dynamics of the gut microbiota [24]. The avian gut bacterial microbiota is dominated by Firmicutes and Proteobacteria phyla, followed by Bacteroidetes and Actinobacteria. They vary in relative abundance depending on the species, sex, age, health status, and environment of the host [24]. However, the composition of the GIT microbiota of wild species, like vultures, remains poorly understood, despite their relevance in pathogen transmission and host health [24,25].

The main aims of this study were to characterize the composition of the gut microbiome of *G. fulvus* vultures in wildlife rehabilitation centers in mainland Portugal and to assess their potential as reservoirs of pathogens relevant to both humans and animals.

## 2. Materials and Methods

### 2.1. Animals

*Gyps fulvus* individuals included in this study were present in two rehabilitation centers: “Centro de Estudos e Recuperação de Animais Selvagens”, located at Castelo Branco, Portugal (CERAS) (n = 7), and “Centro de Recuperação de Animais Selvagens”, located at Santo André (CRASSA) (n = 1) (Table 1).

All animals were fed with rabbits or frozen chicken sourced from intensive farming intended for human consumption, and none had received medication in the last ten days. Additionally, none of the griffon vultures were fed the day before sample collection to prevent regurgitation.

### 2.2. Sample Collection and Handling

On the day of sampling, the animals were moved to individual cleaned and disinfected (bleach solution) enclosures and kept there for at least four hours with free access to water. After, feces were collected during routine examination procedures using sterile swabs and placed in sterile tubes. These fecal samples were distributed into fresh tubes (fresh sample) and into tubes containing an RNA later solution (Invitrogen, Thermo Fisher Scientific, Vilnius, Lithuania) in a proportion of 1:1. Samples were then stored at 4 °C until DNA extraction. Samples were collected from April 2019 to February 2020 in different periods of time, e.g., May/spring, August/summer, and October/early autumn, aiming to detect possible differences in the microbiome related with the different seasons.

All animals were cared for according to the rules given by the current EU (Directive 2010/63/EC) [26] and national (DL 113/2013) [27] legislation and by the competent authority (Direção Geral de Alimentação e Veterinária, DGAV, www.dgv.min-agricultura.pt/portal/page/portal/DGV accessed on 18 November 2024). Only noninvasive samples were collected during routine procedures, according to the regulations of the rehabilitation centers. Trained veterinarians obtained all the samples, following standard routine procedures. No animal experiment has been performed in the scope of this research.

### 2.3. DNA Extraction and Quantification

Fecal samples were weighted to obtain samples ranging between 180 and 220 mg. Then, total bacterial DNA extraction was carried out using the QIAamp^®^ DNA Stool Mini Kit 50 (QIAGEN, Hilden, Germany), following the manufacturer’s instructions.

For DNA concentration and purification, Sera-Mag Carboxylate-4 Modified Magnetic Beads in an OpenWetWare binding buffer were used. Quality control and quantification of the recovered DNA were assessed by fluorimetry (Qubit^®^ 19 v 1.01, Thermo Fisher Scientific, Vilnius, Lithuania).

### 2.4. Amplification and Sequencing of the 16S rRNA Genes

PCR amplification of the V3-V4 hypervariable regions of the bacterial 16S rRNA was performed using two universal primers, 341F (5′-CCTACGGGAGGCAGCAG-3′) and 907R (5′-CCGTCAATTCMTTTGAGTTT-3’), and the following cycling conditions: initial denaturation for 3 min at 94 °C; 30 cycles of denaturation at 94 °C for 20 s; annealing at 45 °C for 30 s; extension at 65 °C for 1 min; and a final extension at 65 °C for 10 min. Then, amplicons were detected by agarose gel electrophoresis using a 1.2% agarose gel stained with Green Safe (NZYtech^®^, Lisbon, Portugal), and the results were visualized by transillumination (ChemiDoc XRS+, Biorad^®^, Hercules, CA, USA).

Afterwards, microbial profiling was performed by the sequencing of the V3V4 16S rDNA gene region [28]. Each sample and sequencing run were carried out on MySeq Illumina sequencing platform. A pair-ended 16S rRNA sequencing library was constructed using the Illumina 16S Sample Preparation Protocol as well as the Nextera XT Index kit, according to the manufacturer’s instructions. The resulting library was then loaded onto a MiSeq platform and sequenced following standard Illumina protocols, generating over 1.9 million reads in total.

### 2.5. Data Analysis and Statistics

Metagenomic data resulting from 16S rRNA sequencing were analyzed using the bioinformatics pipeline Quantitative Insights into Microbial Ecology (QIIME2) for taxonomic classification. Quality control was performed using Debur workflow with the trimming of 150 bps. A sequencing quality score, or Phred quality score, of 20 was employed to assess sequence quality, ensuring a base accuracy of 99%.

Alpha diversity (microbial diversity within a roost) was assessed using Pielou’s evenness index, which was calculated for each group (male vs. female; CERAS vs. CRASSA), followed by the comparison of the variance between the indexes of each group using the Kruskal–Wallis non-parametric test. Beta diversity (microbial diversity between roosts) was analyzed for all groups using the Jaccard similarity index. This analysis produced a Principal Coordinates Analysis (PCoA) plot in which samples with similar compositions were closely clustered. A heatmap representing the different abundances of the different Operational Taxonomic Units (OTUs) for each sample was generated. Comparing the thirty-four more abundant taxa, a phylogenetic tree was established for the sampled individuals [29]. Venn diagrams were produced using InteractiVenn^®^ to illustrate how many shared and exclusive taxa were found between the individuals within the groups. By studying the taxa identified across all samples, human and animal pathogen taxa were also identified.

## 3. Results

It was possible to obtain DNA from all samples obtained (n = 8) with concentrations ranging between 286 ng/µL and 1080 ng/µL, which was further analyzed.

### 3.1. Taxonomic Richness and Relative Abundance of Taxa

A total of 5,078,877 high-quality reads were obtained from the eight individuals’ fecal samples. The reads of the samples were normalized, ensuring a comparable analysis between samples. The demultiplexed per-sample counts were as follows: CER1 (772.849), CER2 (515.871), CER3 (432.555), CER4 (564.429), CER5 (943.217), CER6 (472.943), CER7 (707.465), and CRA1 (669.548).

The fecal microbiota of the *G. fulvus* sampled presented a total of 10 phyla, 16 classes, 25 orders, 47 families, 60 genera, and 34 species (Table 2). All samples contained similar richness in taxa. Sample CRA1 presented the lowest number of identified OTUs at the family, genus, and species levels, and CER3 presented the lowest number of identified OTUs at the order level.

**Figure 1:**
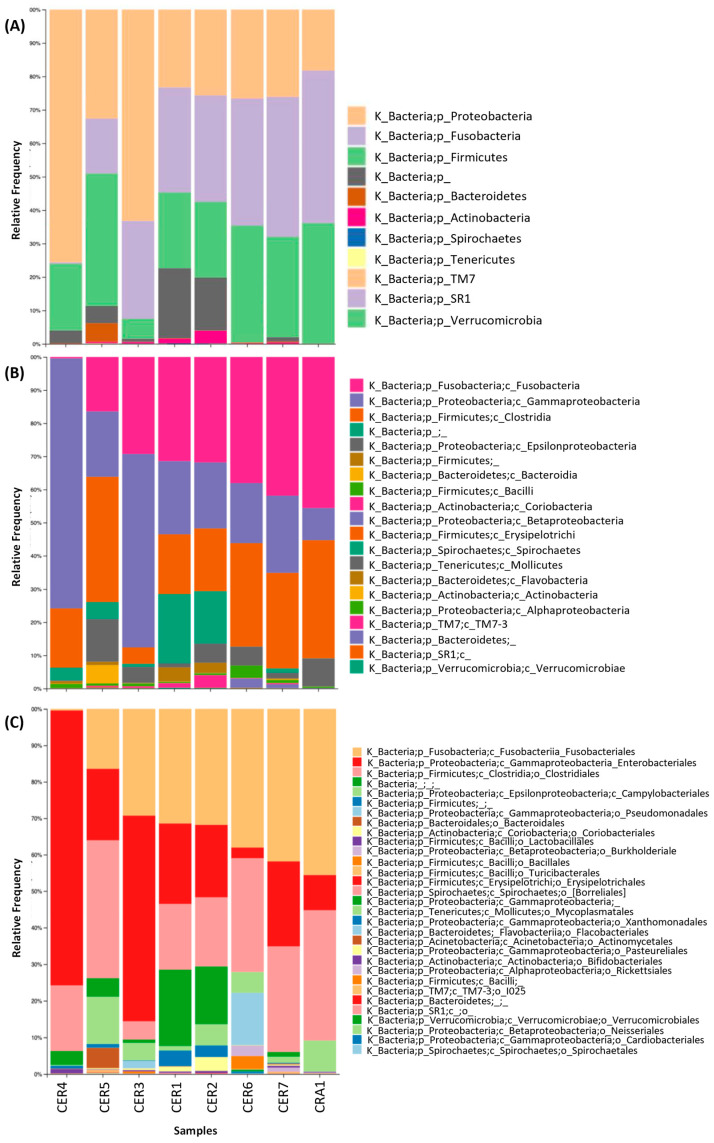
Relative abundance of phyla (**A**), classes (**B**), and orders (**C**) present in each *Gyps fulvus* fecal sample analyzed.

Considering all *G. fulvus* fecal samples, the most abundant phyla identified were Proteobacteria (36.4%) followed by Fusobacteria (29.4%) and Firmicutes (26.5%). The lowest percentages were observed for Bacteroidetes (0.9%), Actinobacteria (0.8%), and SR1/Absconditabacteria phyla (Figure 1A).

Although Proteobacteria was the most abundant phylum on average, Fusobacteria was the most abundant phylum in most samples (CER1, CER2, CER6, CER7, and CRA1). Sample CER4, comprising the highest percentage of Proteobacteria (75.6%) in its composition, presented the lowest rate of Fusobacteria (0.4%). Regarding the classes identified across all tested samples, the most abundant was the Gammaproteobacteria (30.8%), immediately followed by Fusobacteriia (29.4%) and by Clostridia (24.1%) (Figure 1B). In addition, the most abundant orders identified in all samples were Fusobacteriales (29.4%), Enterobacteriales (28.6%), Clostridiales (24.1%), Campylobacteriales (5.1%), and Pseudomonadales (2%) (Figure 1C). It is also important to note that the results revealed an average of 5.4% of unclassified bacteria.

### 3.2. Core Microbiome

*G. fulvus* core microbiome, herein defined as the taxa identified across all fecal samples, comprised six phyla (Actinobacteria, Bacteroidetes, Firmicutes, Fusobacteria, Proteobacteria, and SR1), nine classes (Actinomycetia, Bacteroidia, Flavobacteriia, Bacilli, Clostridia, Fusobacteriia, Beta-, Epsilon-, and Gamaproteobacteria), nine orders (Actinomycetales, Bacteroidales, Lactobacillales, Turcibacteriales, Clostridiales, Fusobacteriales, Campylobacteriales, Enterobacteriales, and Pseudomonadales), twelve families (Porphyromonadaceae, Enterococcaceae, Turcibacteriaceae, Clostridiaceae, Lachnospiraceae, Peptostreptococcaceae, Tissierellaceae, Fusobacteriaceae, Campylobacteraceae, Helicobacteriaceae, Enterobacteriaceae, and Moraxellaceae), five genera (*Turcibacter*, *Clostridium*, *Epulopiscium*, *Peptostreptococcus*, and *Helicobacter*), and one species, *Clostridium perfringens*, which is a potential human and animal pathogen (Supplementary Material—Table S1).

**Figure 2:**
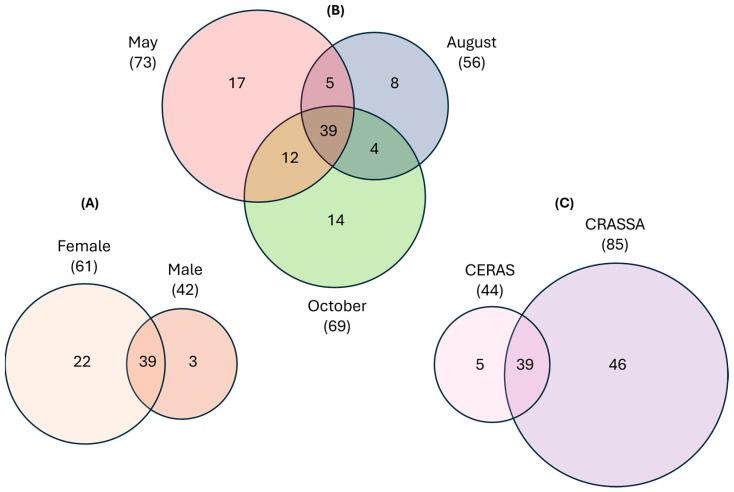
Venn diagrams illustrating the differences between the number of taxa present in *Gyps fulvus* gastrointestinal microbiomes. (**A**) Difference between female and male samples; (**B**) between samples collected in May, August, and October; (**C**) between samples collected in CERAS and CRASSA. Generated with InteractiVenn^®^ (http://www.interactivenn.net/ accessed on 1 October 2024).

Besides the taxa present in the core microbiome, other taxa were also found only at the individual level (exclusive microbiota) or in specific groups, varying according to the different genders (male and female), period of sample collection (May/spring, August/summer, October/early autumn), and location of sample collection (CERAS and CRASSA). The regions in which CERAS and CRASSA are located exhibit distinct ecological traits that may influence vultures’ microbiome. CERAS is located in the interior central region of Portugal near the Spanish border, with a Mediterranean continental climate and vegetation, mountainous terrain, dry vegetation, and raptor-friendly habitats. CRASSA is situated in the coastal Alentejo region in the southwest of Portugal, with a coastal, Mediterranean maritime climate, coastal and estuarine ecosystems, and diverse birdlife. Differences in the composition of the gastrointestinal microbiome were also identified between the three groups (Figure 2).

Samples from females presented 61 taxa, with 22 being exclusive to this group and 39 also found in male microbiome. Samples from males presented only three exclusive taxa, out of a total of 42. The number of taxa (n = 73), including exclusive taxa (n = 17), found in samples collected in May was higher than in those collected in October (n = 69 and n = 14, respectively) and in August (n = 56 and n = 8, respectively). The comparison between results from samples collected in different periods revealed a higher variation between the taxa of samples collected in May and October (12). The sample recovered in CRASSA had a higher number of taxa (n = 85), including exclusive taxa (n = 46), than the samples recovered in CERAS, which presented a total of 44 taxa, with five of them exclusive. Overall, samples from females collected in May showed higher microbiome diversity.

Detailed information on differing gut microbiota taxa between sexes (Supplementary Material—Table S2), the period of sample collection (Supplementary Material—Table S3), and the location of sample collection (Supplementary Material—Table S4) can be found in Supplementary Materials.

### 3.3. Variability Between the Sampled Individuals

**Figure 3:**
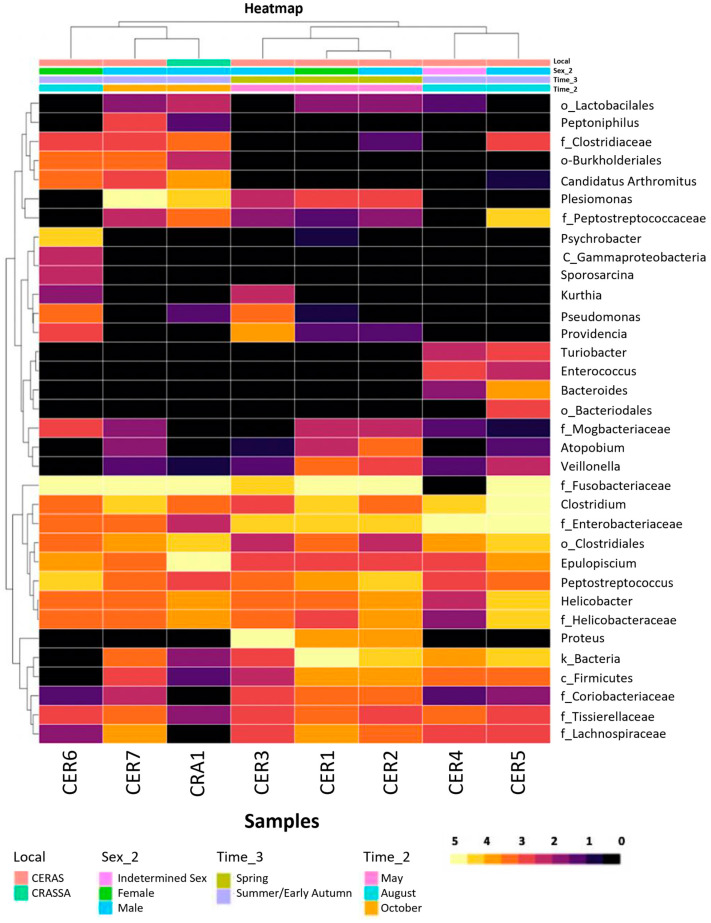
Heatmap of the most abundant thirty-four taxa (normalized log10 abundance of each OTU for each sample) present in the *Gyps fulvus* fecal samples. Different tones correspond to differences in taxa abundance (most to least abundant taxon, lighter (5, yellow) to darker tones (0, black)). Included on the left is a phylogenetic tree of the OTUs and, on top, a cluster tree of the samples based on their similarity regarding these 34 taxa. Samples’ information (location of recovery, sex, and time of collection) is labeled on the bottom.

An OTU heatmap and a phylogenetic and a cluster tree were generated based on the relative frequency of the thirty-four most abundant OTUs within all individuals (Figure 3).

Individuals CER1 and CER2 were clustered together after microbiome characterization, followed by CER3. This cluster was assembled into another cluster formed by CER4 and CER5. These clusters were assembled into a clade. Individuals CRA1 and CER7 were clustered jointly and then with CER6, forming a clade that was closely related to the previous one.

### 3.4. Diversity Analysis of the Griffon Vulture’s GIT Microbiota

**Figure 4:**
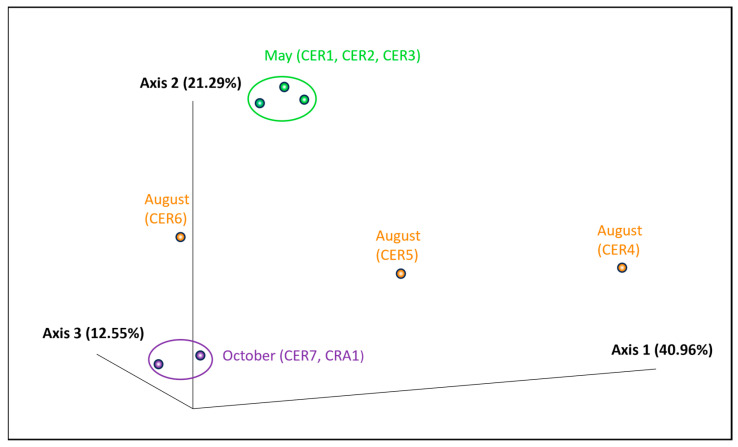
T PCoA plot based on the Jaccard distance matrix, with individuals sampled in May represented in green, those sampled in August in orange, and those sampled in October in purple.

Alpha diversity revealed that there is a higher evenness in the female population when compared to the male population and within the samples collected during spring compared to the ones collected during summer and early autumn. The diversity of the microbiota of the individuals sampled during early autumn was also recognized as being more even when compared with the diversity of the individuals’ samples collected during spring and summer. The Kruskal–Wallis test did not reveal significant differences among the groups since all *p*-values were higher than the levels of significance 0.05 (Supplementary Material—Table S5). Beta diversity was evaluated using the Jaccard distance matrix and can be observed in the PCoA plot (Figure 4).

Individuals sampled in spring (CER1, CER2, and CER3) were clustered together, as were the cohorts sampled in early autumn (CER7 and CRA1). This shows that the microbial community of CER1, CER2, and CER3 individuals is more similar to the one from CER7 and CRA1 than to the one from individuals CER4, CER5, and CER6 collected during summer. In Figure 4, these last animals were found scattered further apart. No clusters were established by the female, male, CERAS, and CRASSA communities, which are more dissimilar from each other’s.

### 3.5. Human and Animal Pathogens

A considerable number of potential human pathogen taxa were found across all samples collected, belonging to fourteen orders, sixteen families, fifteen genera containing five species associated with human diseases (Supplementary Material—Table S6). Specifically, one order (Ricketsiales), four families (Listeriaceae, Alcaligenaceae, Enterobacteriaceae, Borreliaceae), and six genera (*Bacillus*, *Streptococcus*, *Clostridium*, *Neisseria*, *Campylobacter*, and *Treponema*) containing species capable of causing conditions listed as notifiable diseases by the World Health Organization [30], such as anthrax, listeriosis, pneumococcal disease, botulism, tetanus, boutonneuse fever, whooping cough, meningococcal disease, gonorrhea, campylobacteriosis, salmonellosis, cholecystitis, bacteremia, cholangitis, urinary tract infection, traveler’s diarrhea, neonatal meningitis and pneumonia, plague, yersiniosis, shigellosis, syphilis, and Lyme disease.

Regarding animal pathogens, three orders, two families, and six genera containing species that are responsible for notifiable diseases by the World Organization for Animal Health [31] were found in the GIT microbiota of *Gyps fulvus* (Supplementary Material—Table S7). This is concerning, as some are zoonotic. Potentially pathogenic taxa identified included: the order Actinomycetales, which includes the agents responsible for tuberculosis (*Mycobacterium* spp.), paratuberculosis (*M. avium*), and diphtheria (*Corynebacterium diphtheriae*) in several animal species; the order Ricketsiales, associated with cowdriosis (*Ehrlichia ruminantium*); the order Burkholderiales, which includes the agent of equine glanders (*Burkholderia mallei*); the family Alcaligenaceae, responsible for contagious equine metritis (*Taylorella equigenitalis*); the family Enterobacteriaceae, namely *Salmonella* spp., which causes salmonellosis in animals and humans; the genus *Bacillus*, associated with anthrax (*B. anthracis*) in wild and domestic animals; *Clostridium* spp., responsible for blackleg in cattle (*C. chauvoei*); *Campylobacter* spp., which leads to bovine genital campylobacteriosis (*C. fetus*); *Pasteurella* spp., the agent of pasteurellosis, fowl cholera, and bovine hemorrhagic septicemia (*P. multocida*); *Mycoplasma* spp., which can cause bovine peripneumonia (*M. mycoides*), contagious caprine pleuropneumonia (*M. capricolum*), and avian mycoplasmosis (*M. galisepticum*, *M. meleagridis*, *M. synoviae*); and finally the genus *Erysipelothrix* spp., responsible for swine and human erysipelas (*E. rhusiopathiae*) [31].

Besides these agents, other taxa able to cause diseases in other animals, including humans, were also found, namely *Streptococcus* spp., among which *S. suis* is a major swine pathogen worldwide responsible for major financial losses in the swine industry [32]; *C. perfringens*, associated with necrotic enteritis in cows and chickens, enterocolitis in pigs, gastroenteritis in dogs, and necrotizing enterocolitis in horses [33,34]; *C. tetani*, the tetanus agent, which particularly affects horses and sheep [35]; *Campylobacter jejuni*, a frequent colonizer of the poultry intestines without apparent symptoms, potentially leading to the contamination of poultry carcasses and subproducts, with posterior spread to humans, causing gastroenteritis [36]; *Helicobacter* spp., which includes more than 35 species associated with disease on humans and animals [37]; and *Moraxella* spp., which includes relevant agents such as *M. bovis, M. ovis,* and *M. bovoculi*, associated with infectious keratoconjunctivitis, the most common ocular illness in ruminant farms worldwide [38].

Of notice, five potentially pathogenic bacterial species were found in the vultures’ microbiome, namely *C. perfringens*, *C. tetani*, *C. ramosun*, *C. spiroforme*, (Phylum Firmicutes), and *Arcobacter cryaerophilus* (Phylum Proteobacteria). It was also possible to identify bacterial genera, families, and orders that include species associated with diseases in humans and animals; however, as the identification at the species level was not possible, a definite conclusion on the zoonotic and pathogenic potential of these bacteria cannot be achieved.

## 4. Discussion

The three most abundant phyla that constitute the bacterial community of the Griffon vulture’s GIT are all common in a wide range of habitats, including soil and waters [24], and resemble the ones found in other vultures and scavengers [39,40,41]. The *G. fulvus* GIT microbiome is distinct from the one from other birds [42,43], further suggesting the existence of a conserved GIT microbial ecological niche amongst vultures and other scavengers as a result of their similar feeding habits [44]. This agrees with the functional core hypothesis, which stands for a similar GIT functional need in phylogenetically closed animals, in opposition to the core microbiota, which states that the base microbiota is shared among all individuals with no interchangeable taxa for the same functions [44].

Proteobacteria was the most abundant phyla on average due to its high proportion within the GIT microbiome of individuals CER4 (75.6% of its total identified bacteria) and CER3 (63.2%). However, the most abundant phylum present in most samples (five out of eight) was Fusobacteria, which further aligns with previous studies regarding New World vultures, specifically Black Turkey (*Coragyps atratus*) and Turkey Vulture (*Cathartes aura*) [39], and other scavengers, including the American alligator (*Alligator mississippiensis*) [45] and the Tasmanian devil (*Sarcophilus harrisii*) [46]. The selection towards a dominant phylum is possibly correlated to its high functional variability [47,48], as Proteobacteria play an important role in preparing the gut for posterior colonization by obligate anaerobes by consuming oxygen and lowering the environment’s redox potential [49], contributing to the homeostasis of the anerobic environment [48]. Fusobacteria are poorly studied regarding their GIT presence, but their abundance in scavengers can suggest their importance to hosts’ health or nutrient acquisition [41].

The higher proportion of Proteobacteria identified in the samples CER3 and CER4 may be a consequence of an underlying, non-detected medical condition, similarly to what happens in humans due to chronic stress [50]. In these individuals, stress may be a consequence of intra-specific competition [51] and captivity [52]. In fact, captivity can cause weight loss, reproductive suppression, changes in the immune system and in glucocorticoid hormone levels, and can last for months or years [52]. For example, some studies found through a bacterial killing assay that whole blood was less effective at eliminating two *Staphylococcus* species in red knots held in captivity for one year, than in wild birds [53], showing a correlation between the stress caused by captivity and a decreased effectiveness of the immune system [52]. However, changes in the immune system may not be predicted as different species may respond differently to captivity [52], like the house sparrows, which increase *E. coli* elimination after 3 weeks of captivity [54]. These studies substantiate the role of the brain–gut microbiota axis in *Gyps fulvus*, suggesting Proteobacteria as a disruptor of the GIT and in the host’s health [55,56]. A significant overrepresentation of the family Enterobacteriaceae was found in individuals subjected to chronic stress [57], as well as in individuals CER4 (75% of its total GIT microbiome) and CER3 (56% of its total GIT microbiome) from this study. In opposition, CER1 and CER2 individuals showed a lower relative abundance of Proteobacteria. As wild birds are thought to carry a higher proportion of Proteobacteria in their GIT, this may be related with the time period spent in captivity [24]. Wild birds’ gut microbiomes are adapted to diverse and sometimes unpredictable conditions, which often support the presence of Proteobacteria. However, captivity provides a controlled and stable setting, which may lead to microbiome adaptation and a potential reduction in Proteobacteria. Individual CRA1 presented the lowest relative abundance of Proteobacteria, probably because it was a juvenile. The inverted pattern regarding the relative abundance of Fusobacteria and Proteobacteria amongst individuals CER4 and CRA1 may also be a consequence of microbial competition [58].

The presence of *Helicobacter* across all fecal samples aligns with previous studies in which this genus was identified in the GIT of other birds, including non-carnivore or scavenger species [59], and may be indicative of a low pH environment throughout the *G. fulvus* GIT [60]. Low gastric pH maintenance was already supported as one of the defense mechanisms of scavengers, preventing the growth and action of other potential pathogenic bacteria present in decaying flesh [22]. The presence of the Betaproteobacteria class in the *G. fulvus* core microbiome might be a consequence of its ubiquitous contact with aquatic environments [61]. The GIT of California condor (*Gymnogyps californianus*), Black (*Coragyps atratus*) and Turkey vultures (*Cathartes aura*), the most widespread of the New World vultures, and the American Crow (*Corvus brachyrhynchos*) also comprises this family across most sampled individuals [40,62], suggesting that it might play an important role amongst carrion eaters.

The class Clostridia represented 24.1% of the core microbiome and is present in the core gut microbiome of other vultures [62]. *Clostridium* was the most abundant genus from the family Clostridiaceae detected and was already described as present in scavengers such as the Old and New World vultures [62,63]. Of special concern was the identification of the potentially pathogenic *C. perfringens* in high abundance, as previously found in Black and Turkey vultures [62]. We observed no major differences between our results and those from a previous study by Zepeda-Mendoza and collaborators [62]. In that study, the dominant class within Firmicutes was Clostridia, of which the most abundant taxa were *C. perfringens* and *C. botulinum*; among Proteobacteria, the most prevalent taxa were Burkholderiales (from Betaproteobacteria), Epsilonproteobacteria (from the delta/epsilon subdivision), and Enterobacteriales (mainly *Escherichia* from Gammaproteobacteria); and in Fusobacteria, the most abundant species were the potential pathogens *Fusobacterium mortiferum*, *F. varium*, and *F. ulcerans* [62].

The Firmicutes/Bacteroidetes ratio in the GIT of the *G. fulvus* under study was as high as the one previously described for other scavengers, like the Tasmanian devil or the blue fox (*Vulpes lagopus*) [46,64]. A higher ratio was already associated with a more efficient extraction of energy by wildlife species [46]. In Portugal, carrion disposable sites are rare and not often sustained, increasing the need for efficient energy extraction from their occasional feedings. A very low Firmicutes/Bacteroidetes ratio due to high Bacteroidetes counts (6%) was present in individual CER5, probably due to dysbiosis, as previously described in humans [65].

A comparison between Pielou’s alpha diversity indexes revealed that the individuals from the female group and the individuals sampled in spring were the ones with smaller differences in the frequencies of the different OTUs. The higher similarity amongst the individuals sampled in spring can be attributed to the fact that these vultures shared the same enclosure, which may be a relevant source of microbial transfer to their inhabitants, resulting in a shared gut microbiota [66]. The number of observed OTUs was similar between males and females, suggesting that sex has no influence on the diversity of the gut microbiota, probably because of the similar ingestion behaviors between male and female griffon vultures. We hypothesize that the decreased richness identified in the GIT microbiota of the individual CER4 was possibly a consequence of its higher susceptibility to chronic stress, leading to a loss of microbiota diversity [50,67], and, in individual CRA1, it was probably due to this animal being a juvenile, as young birds may have not yet developed a stable GIT [24]. In animals in captivity, chronic stress, triggered by unfamiliar factors like cage confinement, human presence, and artificial lighting, can lead to weight loss, immunosuppression, reproductive failure, and psychological distress [68,69]. Captivity impacts animal species differently [52]—while some adapt, others, such as large-range predators, often experience severe behavioral changes and newborn mortality [70].

The Kruskal–Wallis test showed that sex, location, and time of collection had no statistical influence on the griffon’s gut microbiota. However, it is important to note that the sample size was too low to allow the comparison between the microbiome from animals from different locations, and so conclusions must be drawn carefully. The absence of correlation between animals’ sex and the gut microbiota was previously observed in other scavengers [41] and other vultures [39], and the absence of correlation between the location of sample collection and the gut microbiota was previously detected in *G. fulvus* and the Egyptian vulture (*Neophron percnopterus*) [71].

PCoA allowed us to observe that there was a dissimilarity in the microbial composition of the GIT within and between the female, male, CERAS, and CRASSA groups. Only the individuals sampled in spring and early autumn comprised a more similar intragroup GIT composition, forming clusters. The heatmap further suggested that the time of sampling was the variable that contributed the most to the similarity of the gut microbiome profiles. Therefore, different seasons might lead to gut microbial variations in vultures, probably due to dietary changes depending on the climate, geography, and migratory patterns of the carcasses of the animals they consume, as in bats due to hibernation [72], suggesting the existence of a core microbiome, as observed in other birds, mammals, and humans [24,73,74].

Several potential human and veterinarian pathogens were identified in the *G. fulvus* GIT microbiota, yet scavengers are often resistant to these bacteria and associated toxins, either through antibacterial properties of their blood [75,76,77,78] or through an indigenous gut microbiota that excludes pathogens, as described for the American black vulture (*Coragyps atratus*) [23]. It is likely that these microorganisms were already present in the vultures’ microbiome prior to their arrival at the recovery center, being originated from carcasses or the environment. Several microorganisms were already detected in the digestive tract of *C. atratus*, like *Actinomyces bovis*, *Lactobacillus cellobiosus*, and *Micrococcus luteus*, having an ecological role in terms of population auto-control and as an environmental barrier in the digestive tract [23]. Recently, Espunyes et al. [79] have identified Eurasian griffon vultures as carriers of zoonotic *Salmonella* and *Campylobacter* bacteria. Additionally, Nesic et al. [80] identified *Mycobacterium avium* in a dead vulture.

The contamination of waters and soils by griffon vultures’ feces can pose several risks, particularly pathogen transmission, nitrogen, and phosphorus from vulture feces overload, chemical contaminants like heavy metals, antibiotic resistance, and water quality. To mitigate these risks, it is important to maintain clean water sources, to monitor and manage vulture populations, and ensure the proper disposal of livestock carcasses to reduce vulture scavenging near human populations. More research is needed to understand the real extent and effects of these risks, particularly pathogen transmission to the environment, humans, and other animals [18].

The small sample size and the uneven distribution of animals across the groups under study introduced some limitations to this study, particularly in terms of data comparison and drawing final conclusions. Additionally, since the sex of one individual could not be identified, this limited the understanding of the relationship between gut microbiome composition and sex-based differences.

## 5. Conclusions

The impact of the GIT microbiome on the host’s health has been proven to be of great importance, and even more so in vultures, which, due to their scavenging diet, are in close contact with pathogenic microorganisms from decomposing meat. This study contributed to the characterization of the recovering *G. fulvus* gut microbiome in mainland Portugal, as it has revealed that its constituents are highly similar to ones from other wild vultures and scavengers. It suggests that captivity-associated stress does not induce large shifts in the bacterial GIT composition of recovering griffon vulture individuals. Still, it was possible to observe that, in a small percentage of the animals, captivity-associated chronic stress could indeed lead to GIT microbiota dysbiosis and reduced diversity overall.

Furthermore, no significant differences were noted between the GIT microbiota profiles of individuals recovering in different locations, and no sex-modulated changes were identified in the gut microbiome of the animals under study. Still, our data suggest possible age-associated differences in the *G. fulvus* GIT microbiome, as younger animals revealed a less diverse and more transient gut microbiome profile. Also, griffon vultures sampled during the same period showed a more similar gut composition, suggesting a seasonal change in their gut microbiome.

The fact that the composition of the gut microbiome of griffon vultures is so similar to that of other vultures and scavengers suggests a well-conserved functional gut microbiome, possibly due to their similar and unique scavenging diet and their functional needs. This way, our study agrees with the functional core hypothesis.

It was also possible to find evidence that griffon vultures are potential reservoirs of human and animal pathogens, including *C. perfringens* and *Mycobacterium* spp., as well as other species capable of causing high priority diseases. Further studies are crucial to understand the dynamics of pathogen shedding and persistence in vulture feces to assessing the extent of environmental contamination.

## Figures and Tables

**Table 1:** Characterization of the *Gyps fulvus* sampled in this study, including the period and location of sample collection.

Sample ID	Sex	Age	Period of Collection	Rehabilitation Center
CER1	Female	Adult	May/spring	CERAS
CER2	Male	Adult	May/spring	CERAS
CER3	Male	Adult	May/spring	CERAS
CER4	Indetermined	Adult	August/summer	CERAS
CER5	Male	Adult	August/summer	CERAS
CER6	Female	Adult	August/summer	CERAS
CER7	Male	Adult	October/early autumn	CERAS
CRA1	Male	Juvenile	October/early autumn	CRASSA

**Table 2:** Taxonomic richness of *Gyps fulvus* microbiota, showing the number of each taxonomic level identified in each sample and the total number of OTUs identified.

Taxonomic Level	CER1	CER2	CER3	CER4	CER5	CER6	CER7	CRA1	Total
Phylum	7	7	6	9	8	8	8	8	10
Class	11	12	11	14	13	13	13	11	16
Order	16	17	14	20	17	16	18	15	25
Family	28	26	25	33	26	36	29	24	47
Genus	30	31	28	30	22	31	22	20	60
Species	14	14	15	17	14	17	13	7	34

## Data Availability

All the data presented in this study are included in the manuscript and Supplementary Materials.

## Supplementary Materials

The following supporting information can be downloaded at https://www.mdpi.com/article/10.3390/vetsci11120622/s1, Table S1: Taxa Identified in fecal samples comprising the core microbiota of the Recovering *Gyps fulvus* in Portugal; Table S2: Differing gut microbiota taxa between the *G. fulvus* of both sexes. Exclusive female taxa (orange) and exclusive male taxa (blue); Table S3: Differing gut microbiota taxa between the *G. fulvus* individuals sampled in May (green), August (orange), and in October (blue); Table S4: Differing gut microbiota taxa between the *G. fulvus* individuals sampled in CERAS and in CRASSA. Exclusive CRASSA taxa (orange) and exclusive CERAS taxa (blue); Table S5. *p*-value results from the Kruskal–Wallis pairwise tests with all variables analyzed; Table S6. Set of human pathogens containing taxa found in the samples of recovering griffon vultures in Portugal. Marked with an * and in orange, are the taxa to which pathogens capable of causing conditions listed as mandatory communicable diseases in Portugal; Table S7. Set of animal pathogens containing taxa found in the samples of recovering griffon vultures in Portugal. Marked with an * and in orange are the taxa to which pathogens capable of causing conditions are listed as mandatory communicable diseases in Portugal.
