# Supplementary material for: Exploring the Gastrointestinal Microbiome of Eurasian Griffon Vultures (Gyps fulvus) Under Rehabilitation in Portugal and Their Potential Role as Reservoirs of Human and Animal Pathogens
Source: Vet Sci. 2024 Dec 4;11(12):622. doi: 10.3390/vetsci11120622 (PMC11680295; doi:10.3390/vetsci11120622)
Supplement: Supplementary file 1 [file vetsci-11-00622-s001.zip › vetsci-3285447-supplementary.pdf]

SUPPLEMENTARY DATA

Table S1: Taxa Identified in fecal samples comprising the core microbiota of the Recovering *Gyps fulvus* in Portugal.

**Phylum**	**Class**	**Order**	**Family**	**Genus**	**Species**
Actinobacteria or Actinomycetota	Actinobacteria or Actinomycetia	Actinomycetales			
Bacteroidetes	Bacteroidia	Bacteroidales	Porphyromonadaceae		
	Flavobacteriia				
Firmicutes	Bacilli	Lactobacillales	Enterococcaceae		
		Turcibacteriales	Turcibacteriaceae	*Turcibacter*	
	Clostridia	Clostridiales	Clostridiaceae	*Clostridium*	*Clostridium perfringens*
			Lachnospiraceae	*Epulopiscium*	
			Peptostreptococcaceae	*Peptostreptococcus*	
			Tissierellaceae		
Fusobacteria	Fusobacteriia	Fusobacteriales	Fusobacteriaceae		
Proteobacteria	Betaproteobacteria				
	Epsilonproteobacteria	Campylobacteriales	Campylobacteraceae		
			Helicobacteriaceae	*Helicobacter*	
	Gammaproteobacteria	Enterobacteriales	Enterobacteriaceae		
		Pseudomonadales	Moraxellaceae		
SR1					

Table S2: Differing gut microbiota taxa between the *G. fulvus* of both sexes. Exclusive female taxa (orange) and exclusive male taxa (blue).

**Phylum**	**Class**	**Order**	**Family**	**Genus**	**Species**
Actinomycetota	Coriobacteriia	Coriobacteriales	Coriobacteriaceae	*Atopobium*	
Bacteroidetes	Flavobacteriia	Flavobacteriales	Flavobacteriaceae		
	Bacteroidia	Bacteroidales	Porphyromonadaceae	*Porphyromonas*	*P. endontalis*
	Bacilli	Bacillales	Planococcaceae		
		Lactobacillales	Enterococcaceae	*Vagococcus*	*V. salmoninarum*
		Turcibacteriales	Turcibacteriaceae	*Turcibacter*	
Firmicutes	Clostridia	Clostridiales	Clostridiaceae	*Clostridium*	*C. paraputrificum*
			Peptostreptococcaceae	*Peptostreptococcus*	*P. anaerobius*

			Peptococcaceae	*Peptococcus*
			Veillonellaceae	
			Ruminococcaceae	
			Mogibacteriaceae	
Proteobacteria	Gammaproteobacteria	Enterobacteriales	Enterobacteriaceae	*Providencia*
		Pseudomonales	Moraxellaceae	*Moraxella*
				Psychrobacter
				Pseudomonas
		Xantomonadales	Xanthomonodaceae	*Wohlfahrtiimonas*

Table S3: Differing gut microbiota taxa between the *G. fulvus* individuals sampled in May (green), August (orange) and in October (blue).

**Phylum**	**Class**	**Order**	**Family**	**Genus**	**Species**
Actinomycetota	Coriobacteriia	Coriobacteriales	Coriobacteriaceae	*Atopobium*	
Bacteroidetes	Bacteroidia	Bacteroidales	Porphyromonadaceae	*Porphyromonas*	*P. endontalis*
			Paraprevotellaceae	*Prevotella*	
	Flavobacteriia	Flavobacteriales	Weeksellaceae		
			Flavobacteriaceae	*Bergeyella*	*B. zoohelcum*
Firmicutes	Bacilli	Bacillales			
		Lactobacillales	Enterococcaceae	*Vagococcus*	*V. salmoninarum*
				Enterococcus	
			Aerococcaceae		
			Streptococcaceae	*Streptococcus*	
	Clostridia	Clostridiales	Clostridiaceae	*Candidatus arthromitus*	
				Clostridium	*C. ramosum*
					C. paraputrificium

			Peptococcaceae		
			Ruminococcaceae		
			Tissierellaceae	*Peptoniphilus*	
Proteobacteria	Betaproteobacteria	Burkholderiales			
		Neisseriales	Neisseriaceae	*Neisseria*	*N. shayegani*
	Epsilonbacteria	Campylobacterales	Campylobacteraceae	*Campylobacter*	*C. rectus*
	Gammaproteobacteria	Enterobacterales	Morganellaceae	*Proteus*	
				Providencia	
		Pasteurellales	Pasteurellaceae	*Pasteurella*	
		Pseudomonales	Moraxellaceae	*Moraxella*	
			Pseudomonadaceae	*Pseudomonas*	
Tenericutes	Mollicutes	Mycoplasmatales	Mycoplasmataceae		
	Erysipelotrichia	Erysipelotrichales	Erysipelotrichaceae		

Table S4: Differing gut microbiota taxa between the *G. fulvus* individuals sampled in CERAS and in CRASSA. Exclusive CRASSA taxa (orange) and exclusive CERAS taxa (blue).

**Phylum**	**Class**	**Order**	**Family**	**Genus**	**Species**
Actinomycetota	Actinomycetia	Actinomycetales	Propionobacteriaceae	*Propionobacterium*	*P. acnes*
	Coriobacteriia	Coriobacteriales	Coriobacteriaceae		
Bacteroidetes	Bacteroidia	Bacteroidales	Porphyromonadaceae	*Porphyromonas*	
			Paraprevotellaceae	*Prevotella*	
	Flavobacteriia	Flavobacteriales			
Firmicutes	Bacilli	Bacillales	Listeriaceae	*Brocothrix*	
		Lactobacillales	Aerococcaceae		
			Carnobacteriaceae	*Carnobacterium*	
			Enterococcaceae	*Vagococcus*	
			Streptococcaceae	*Streptococcus*	
		Turcibacteriales	Turcibacteriaceae	*Turcibacter*	
	Clostridia	Clostridiales	Clostridiaceae	*Candidatus Arthromitus*	
				Clostridium	*C. paraputrificum*

					C. tetani
			Peptococcaceae		
			Peptostreptococcaceae	*Peptostreptococcus*	*C. anaerobius*
			Veillonellaceae	*Veillonella*	
			Mogibacteriaceae		
			Tissierellaceae	*Peptoniphilus*	
Proteobacteria	Betaproteobacteria	Burkholderiales			
		Neisseriales	Neisseriaceae	*Neisseria*	*N. cinerea*
	Epsilonproteobacteria	Campylobacteriales	Campylobacteraceae	*Arcobacter*	*A. cryaerophilus*
	Gammaproteobacteria	Enterobacteriales	Enterobacteriaceae	*Plesiomonas*	*P. shigelloides*
		Pasteurellales			
		Pseudomonales	Moraxellaceae	*Moraxella*	
				Pseudomonas	
TM7	TM7-3	I025	RS-045		
Tenericutes	Mollicutes	Mycoplasmatales	Mycoplasmataceae		

Table S5. *p*-value results from the Kruskal–Wallis pairwise tests with all variables analyzed.

**Variables analyzed**		***p*-value**
Spring	summer-early autumn	0.456057
May	August	0.512691
	October	0.563703
August	October	0.563703
Indeterminate sex (CER4)	Female	0.220671
	Male	0.143235
Female	Male	0.698535
CERAS	CRASSA	0.827259

Table S6. Set of human pathogens containing taxa found in the samples of recovering griffon vultures in Portugal**.** Marked with an * and in orange, are the taxa to which pathogens capable of causing conditions listed as mandatory communicable diseases in Portugal.

**Order**	**Family**	**Genus**	**Species**	**Associated diseases in humans**
Actinomycetales				

Bacillales	Bacillaceae	*Bacillus**		Anthrax (*B. anthracis*)
	Listeriaceae*			Listeriosis (*Listeria monocytogenes*)
Lactobacillales	Streptococcaceae	*Streptococcus**		Pneumococcal disease (*S. pneumoniae*)
Clostridiales	Clostridiaceae	*Clostridium**		Botulism (*C. botulinum*)
			C. perfringens	Gastrointestinal infections, myonecrosis, necrotizing enteritis, septicemia
			C. tetani *	Tetanus
			C. ramosum	Gastrointestinal infections
			C. spiroforme	
Ricketsiales*				Boutonneuse fever (*Rickettsia conorii*)
Burkholderiales	Alcaligenaceae*			Whooping cough (*Bordetella pertussis*)
Neisseriales	Neisseriaceae	*Neisseria**		Meningococcal disease (*N. meningitidis*); Gonorrhea (*N. gonorrhoea*)
Campylobacteriales	Campylobacteraceae	*Arcobacter*	*A. Cryaerophilus*	Gastrointestinal infections
		*Campylobacter**		Campylobacteriosis (*Campylobacter* spp.)
	Helicobacteriaceae	*Helicobacter*		Gastritis, gastric ulcers, MALT-lymphoma (*H. pylori*)
Enterobacteriales	Enterobacteriaceae*			Salmonellosis (*Salmonella spp.);* Cholecystitis, Bacteremia, Cholangitis, Urinary Tract Infection, Traveler's Diarrhea, neonatal meningitis and pneumonia (*Escherichia coli* STEC or VTEC13); Plague *(Yersinia pestis);* Yersiniosis *(Yersinia enterocolitica);* Shigellosis (*Shigella spp.*)
	Morganellaceae	*Morganella* *Providencia*		
Pasteurellales	Pasteurellaceae	*Pasteurella*		Pasteurella: chronic abscesses on the face or extremities (*P. multocida*)
Pseudomonales	Moraxellaceae	*Acinetobacter*		
		Moraxella		

Xantomonadales	Xanthomonodaceae	*Wohlfahrtiimonas*		
Spirochaetales	Spirochaetaceae	*Treponema**		Syphilis (*T. pallidum*)
Borreliales	Borreliaceae*			Lyme Disease (*Borrelia burgdorferi*)
Mycoplasmatales	Mycoplasmataceae	*Mycoplasma*		
Erysipelotrichales	Erysipelotrichaceae	*Erysipelothrix*		Localized and widespread cutaneous lesion (erysipeloid), and septicemia often associated with endocarditis (*E. rhusiopathiae*)

Table S7. Set of animal pathogens containing taxa found in the samples of recovering griffon vultures in Portugal**.** Marked with an * and in orange are the taxa to which pathogens capable of causing conditions are listed as mandatory communicable diseases in Portugal.

**Order**	**Family**	**Genus**	**Species**	**Associated diseases in humans**
Actinomycetales*				Tuberculosis (*Mycobacterium spp.*)<br>Paratuberculosis (*Mycobacterium avium* subsp. *paratuberculosis*)<br>Diphtheria (*Corynebacterium diphtheriae*)
Bacillales	Bacillaceae	*Bacillus**		Anthrax (*B. anthracis*)
	Listeriaceae			
Lactobacillales	Streptococcaceae	*Streptococcus*		Septicemia, pneumonia, endocarditis, arthritis, and meningitis (*S. suis*)
Clostridiales	Clostridiaceae	*Clostridium**	*C. perfringens*	Intestinal diseases in cows, chickens, pigs, dogs, and horses
			C. tetani	Spastic paralysis in horses, sheep, and less in cattle, dogs, and cats
			C. ramosum	Gastrointestinal disorders
			C. spiroforme	Enteric disease in rabbits
				Symptomatic anthrax (*C. chauvoei*)
Ricketsiales*				Cowdriosis (*Ehrlichia ruminantium*)<br>Diseases in livestock and dogs (*Rickettsia* spp.)
Burkholderiales*	Alcaligenaceae*			Equine glanders (*Burkholderia mallei*)<br>Contagious equine metritis (*Taylorella equigenitalis*)
Neisseriales	Neisseriaceae	*Neisseria*		

Campylobacteriales	Campylobacteraceae	*Arcobacter*	*A. cryaerophilus*	Gastrointestinal infections
		*Campylobacter**		Bovine genital campylobacteriosis (*C. fetus*).
	Helicobacteriaceae	*Helicobacter*		Abortion in sheep (*Helicobacter* spp.)
Enterobacteriales	Enterobacteriaceae*			Salmonellosis (*Salmonella spp.)*<br>Sheep and goat's salmonellosis (*S.* Abortusovis)<br>Avian salmonellosis (S. Pullorum, *S.* Gallinarum, *S. arizonae*)
	Morganellaceae	*Morganella*<br>*Providencia*		
Pasteurellales	Pasteurellaceae	*Pasteurella**		Pasteurellosis (*Pasteurella* spp.)<br>Avian cholera<br>Bovine hemorrhagic septicemia (*P. multocida*)
Pseudomonales	Moraxellaceae	*Acinetobacter*		
		Moraxella		Infectious keratoconjunctivitis in ruminant farms (*M. bovis, M. ovis, M. bovoculi*)
Xantomonadales	Xanthomonodaceae	*Wohlfahrtiimonas*		
Spirochaetales	Spirochaetaceae	*Treponema*		
Borreliales	Borreliaceae			
Mycoplasmatales	Mycoplasmataceae	*Mycoplasma**		Contagious bovine peripneumonia (*M. mycoides* subsp. *mycoides*)<br>Contagious caprine pleuropneumonia (*M. capricolum* subsp. *capripneumoniae*)<br>Avian mycoplasmosis (*M. galisepticum M. meleagridis, M. synoviae*)
Erysipelotrichales	Erysipelotrichaceae	*Erysipelothrix**		Swine erysipelas (*E. rhusiopathiae*), Erysipelas in farmed turkeys, chickens, ducks and emus; polyarthritis in sheep and lambs
